# Methods and computational tools to study eukaryotic cell migration *in vitro*

**DOI:** 10.3389/fcell.2024.1385991

**Published:** 2024-06-03

**Authors:** Elvira Toscano, Elena Cimmino, Fabrizio A. Pennacchio, Patrizia Riccio, Alessandro Poli, Yan-Jun Liu, Paolo Maiuri, Leandra Sepe, Giovanni Paolella

**Affiliations:** ^1^ Department of Molecular Medicine and Medical Biotechnology, Università Degli Studi di Napoli “Federico II”, Naples, Italy; ^2^ CEINGE Biotecnologie Avanzate Franco Salvatore, Naples, Italy; ^3^ Laboratory of Applied Mechanobiology, Department of Health Sciences and Technology, Zurich, Switzerland; ^4^ Axxam S.p.A., Milano, Italy; ^5^ Institutes of Biomedical Sciences, Fudan University, Shanghai, China

**Keywords:** cell migration, cell tracking, trajectory analysis, chemotaxis, durotaxis

## Abstract

Cellular movement is essential for many vital biological functions where it plays a pivotal role both at the single cell level, such as during division or differentiation, and at the macroscopic level within tissues, where coordinated migration is crucial for proper morphogenesis. It also has an impact on various pathological processes, one for all, cancer spreading. Cell migration is a complex phenomenon and diverse experimental methods have been developed aimed at dissecting and analysing its distinct facets independently. In parallel, corresponding analytical procedures and tools have been devised to gain deep insight and interpret experimental results. Here we review established experimental techniques designed to investigate specific aspects of cell migration and present a broad collection of historical as well as cutting-edge computational tools used in quantitative analysis of cell motion.

## Introduction

Cells are dynamic entities. They keep exploring the surrounding environment and continuously change their shape reacting to biochemical and physical external stimuli. Individual movements of isolated cells, as well as coordinated motion of groups of cells, generally referred to as cell migration, have been thoroughly studied in recent years. Cell displacement primarily relies on cell polarization, the asymmetric distribution of cytoskeletal cellular components, that leads to cell body orientation in space, and finally, cell movement.

A relevant feature of cell migration studies, especially when conducted via live cell microscopy, is the need to analyse a large number of cells with the consequent generation of consistent to extensive datasets. Computational tools are then necessary for extracting quantitative information from the images and interpreting the obtained results.

Here we present an overview of experimental methods, used to investigate specific aspects of cell migration *in vitro* together with a compilation of “state of the art” computational tools, developed to quantitatively analyse and interpret experimental results.

### Cell movement dynamics and regulation

Cell movement depends on a variety of variables, such as the cell type, the chemical and physical culture conditions, medium composition and nutrients availability, cell-substrate and cell-cell interactions. In absence of external cues, cells tend to randomly move by units to tens of micro-meters, depending on cell type, as if exploring all possible directions (Lauffenburger and Horwitz, 1996). In doing so, they typically show a sort of resistance to abrupt directional changes (Maiuri et al., 2012; Toscano et al., 2022; Amiri et al., 2023). Indeed if observed at a sufficiently short time scale, for example, in the order of minutes, cells appear to move ballistically, while at larger time scales (tens of minutes or hours), they clearly move randomly. Essentially, they show a tendency to maintain their previous direction, as change involves membrane and cytoskeletal reorganisations and cost energy. Both *in vivo* and *in vitro*, cells can change their locomotion properties, when exposed to chemical or physical stimuli produced by chemical gradients, drug treatments, or events such as a wound inflicted in a cell monolayer.

In the prototype of locomotion which is referred to as mesenchymal, the overall movement may be schematically described as a cycle: a cell extends a protrusion at the leading edge, establishes new adhesions with the substratum at the front, then performs a forward movement of its nucleus and body (traction), and finally, detaches the adhesions at the rear and retracts its tail (Gauthier and Roca-Cusachs, 2018; Garcia-Arcos et al., 2019). For mesenchymal migration, levels of cell-substratum adhesiveness is an important determinant of cell migration speed, with maximum migration efficiency found at intermediate adhesion level (DiMilla et al., 1993; Palecek et al., 1997). In poorly adherent cells, in fact, cell speed is apparently limited by the ability to form attachments at the cell front. On the other hand, high cell-substrate adhesion tends to limit cell speed by reducing the rate of cell detachment from the substratum. Impaired detachment and/or tail retraction may decrease movement rate in cells, such as cultured fibroblasts, which tend to be strongly adherent, have an extended tail and leave behind a trail of cytoplasmic fragments as they move (Padhi et al., 2021). Overall, cell displacement is the final result of a series of highly coordinated events led by the polarization of the cell body according to an axis oriented along the direction of motion. Moreover, it requires continuous cytoskeleton rearrangements that must be coordinated both in space and time to generate productive movement. Actin filaments are one of the main components of the cytoskeleton and are double helical polymers of globular subunits aligned head-to-tail. The filaments have an intrinsic molecular polarity: one end, the fast growing one, is called the barbed end (or plus ends); the other one is the pointed end. Actin cytoskeleton organisation is crucial in starting and stabilising the asymmetric distribution of polarity components within the cell. During early events of cell polarization, spatial distribution of filamentous F-actin changes: it loses the circular symmetry typically observed around the perinuclear cell rim, and it concentrates in specific regions at the cell periphery to support and favour the formation of highly dynamic protrusive structures. Actin filaments are there organised with their barbed ends oriented in the direction of the protrusion, while the growing ones point towards the cell body. The simplest structures they form are filopodia, which are long fingers that can extend tens of microns. Lamellipodia, instead, are thin protrusive sheets that dominate the leading edges of cultured fibroblasts and other motile cells. The characteristic rufflings observed at the leading edges of fibroblasts are due to lamellipodia that lift up off the substrate and move backwards. These aspects were effectively addressed in the following readings (Cooper and Schafer, 2000; Pollard and Borisy, 2003; Burnette et al., 2011; Beta et al., 2023).

**FIGURE 1:**
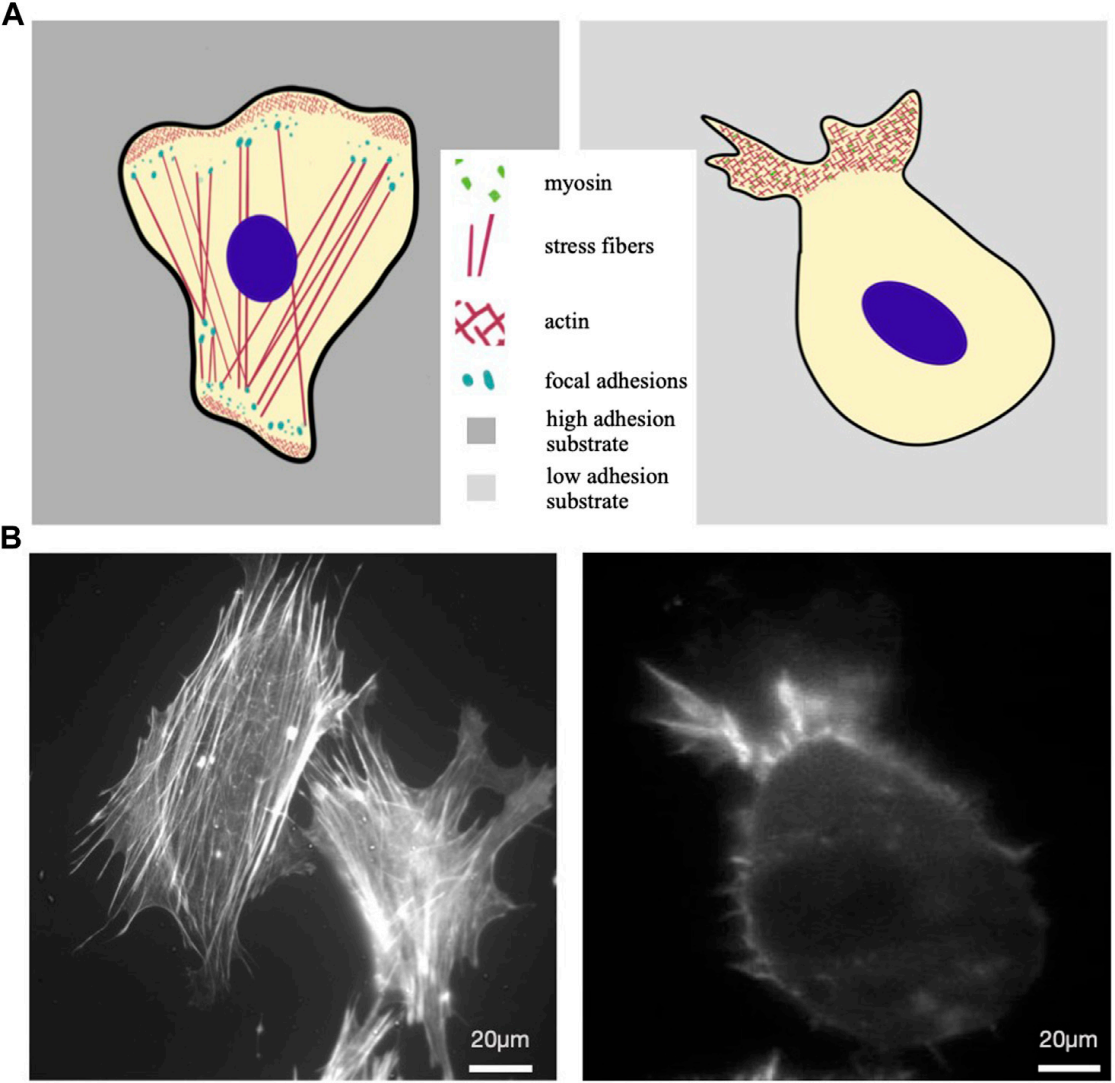
Mesenchymal vs amoeboid migration. **(A)** Sketches of a mesenchymal (left) and an amoeboid cell (right). The first is characterised by irregular shape, numerous actin stress fibers within the cytoplasm that end in focal adhesions; at the leading edge actin-rich structures form typical protrusions, such as lamellipodia and filopodia, driving cell displacement on flat substrates. Cell moving in ameboid mode is illustrated on the right as characterized by a slightly round shape, absence of stress fibers and weaker cell-substrate adhesion, with short actin microfilaments and myosin II committed in the retrograde flow at the leading edge. **(B)** Actin cytoskeleton of a typical mesenchymal cell (MEF, mouse embryonal fibroblast) (left) and RPE1 (Human retinal Pigment epithelial-1 cells) under confinement and low adhesion (right), respectively stained with Phalloidin-TRITC and Lifeact-mCherry.

An alternative locomotion strategy cells may use is the so-called amoeboid migration, which is typical of immune cells. It is based on friction between the cell body and the surrounding space and relies on cell contractility and ability to undergo extensive cellular deformations. This kind of locomotion does not involve cell adhesion and is more efficient, allowing cells to move typically one order of magnitude faster than in mesenchymal motion (DiMilla et al., 1993). A cell moving mesenchymally was compared with one moving exploiting amoeboid motion in Figure 1, where main structural cytoplasmic elements were illustrated in panel a, while the actin cytoskeleton organization of real cells are reported in b. Interestingly, cells that typically move mesenchymally, cultured in low adhesion conditions and subjected to cell body confinement, can switch to ameboid-like migration (Figure 1A). This is consistent with the idea that ameboid locomotion is an ancestral motion strategy, that cells are still able to revert to, whenever necessary for survival (Liu et al., 2015; Venturini et al., 2020; Wang et al., 2023).

In moving cells, morphological changes of the cell body are associated with and depend on corresponding changes at the molecular level (Lauffenburger and Horwitz, 1996). Many cellular components and signalling pathways are involved or partially impact on cell movement, including energy metabolism, cytoskeletal and other structural molecules, membrane receptors and signal transduction pathways. A large number of studies have been focused on one or more of these aspects, including some recently published review articles (Ridley et al., 2003; Yamada and Sixt, 2019; SenGupta et al., 2021; Merino-Casallo et al., 2022; Pawluchin and Galic, 2022). So, since it is not the scope of this review to provide an in-depth description of molecular determinants of eukaryotic cell motion, we are reporting here a very short introduction to the principal molecules and pathways involved. Actin cytoskeleton changes are typically triggered by adhesion processes but play also a role when cell adhesion is not involved, as in amoeboid movement. Actin filaments are mainly arranged in two distinct kinds of structure: linear and branched. The first is typical of filopodia and actin stress fibres and depends on formins. The latter depends on Arp2/3 and constitutes the forest of interconnected branched actin filaments at the front of cell lamellipodia (Cooper and Schafer, 2000; Pollard and Borisy, 2003; Stradal and Scita, 2006; Yang and Svitkina, 2011; Schaks et al., 2019; Zimmermann and Kovar, 2019; Kadzik et al., 2020).

All processes regulating adhesion-dependent mesenchymal cell motility, including cell protrusion, cell retraction, cell-matrix adhesion, polarised exocytosis and polarised vesicle trafficking, are spatiotemporally controlled by specific intracellular signalling pathways and by different intra/extra cellular factors. Among the different players involved in these processes, Rho GTPases (Cdc42, Rac, and Rho) exert a central role, funnelling signals from the extracellular environment to downstream components that modulate cell motility. Cdc42 and Rac promote F-actin assembly and edge protrusion, whereas Rho activation triggers myosin light chain (MLC) phosphorylation, causing myosin-based cell contraction. Rho is also a Rac antagonist and promotes F-actin polymerization through formins (Nobes and Hall, 1995; Etienne-Manneville, 2004; Hall, 2012; Kühn and Geyer, 2014). Molecules and pathways activated by Ras-GTP also play a fundamental role in controlling cell migration: PI3K, for example, accumulates at the cell protruding edge and is implicated in the regulation of actin polymerization and formation of lamellipodia, through its activity on the GTP-binding Rac protein (Weiger et al., 2010; Campa et al., 2015). MAPK/ERK signalling also regulates cell movement, by controlling the expression of genes associated with lamellipodia formation and tumour invasion. It regulates, directly or indirectly, the expression and function of factors such as SNAI2/Slug, TWIST1 and ZEB1/2, thus driving epithelial to mesenchymal transition (EMT) and inducing a pro-motile and pro-invasive cell state (Shin et al., 2010; Xiong et al., 2012; Zhou et al., 2012; Kurihara et al., 2015). Through phosphorylation of substrates such as myosin light chain kinase (MLCK) or Ca^++^-activated calpain, ERK regulates protrusion formation and cell retraction, cell-matrix adhesion and exocytosis, thus controlling coordinated and directional movement (Glading et al., 2004; Sepe et al., 2013). Calcium concentration is indeed an important regulator of both actin polymerization and cell contractility and has therefore a central role in modulating cell migration. In moving cells, calcium concentration is asymmetrically distributed, being higher at the cell rear than at the cell front. This asymmetry, along with localised calcium fluctuations, regulates activation of different intracellular mediators (Wells et al., 2005; Chan et al., 2010; Cortesio et al., 2011).

### Experimental methods for studying cell migration

**FIGURE 2:**
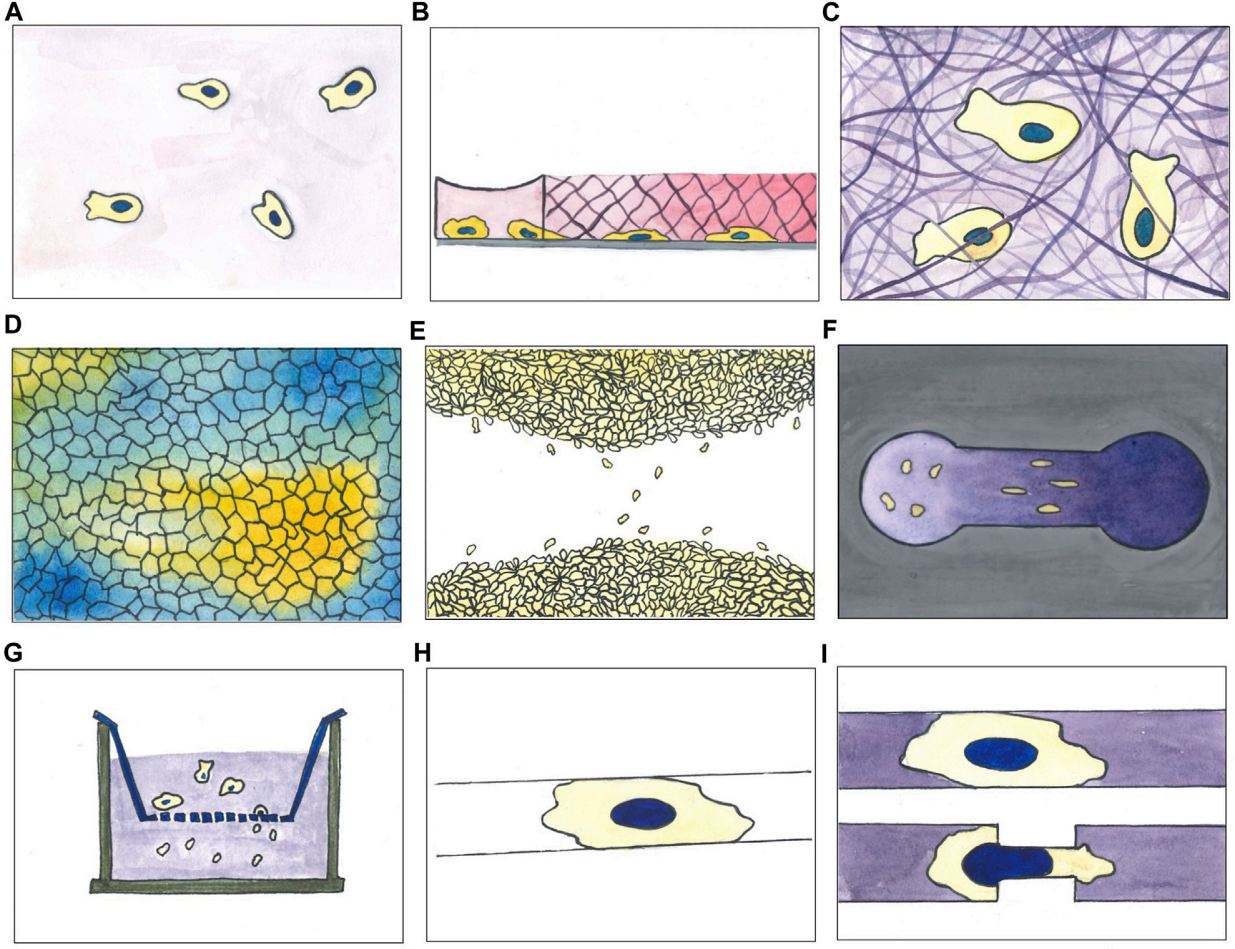
A selection of experimental assays used for studying cell migration *in vitro*. **(A)** 2D movement on culture surface; **(B)** under agarose migration assay (cells initially plated in a reservoir invade the agarose pad moving below it following a chemoattractant gradient, here represented by a pink gradient); **(C)** migration through matrix (cells embedded in a matrix moving inside it; matrix can be of different composition varying in stiffness and pores size); **(D)** collective cell migration with colour code indicating directionality degree of migrating cell population (cluster of cell moving in the same direction are depicted with the same colour); **(E)** wound healing (scratch) assay; **(F)** Zigmond chemotaxis chamber; **(G)** transwell assay; **(H)** micropatterning of adhesive molecules on non-adhesive surfaces (cells plated on this kind of devices spread on the suitable area and move according to the imposed constrains); **(I)** microfluidic-based migration assay within channels combined with constrictions (cells moving inside straight channels or channels with later constrictions).

Different methods have been used to evaluate movement in cultured cells. Many of the involved techniques have been reviewed in depth by Kramer and colleagues (Kramer et al., 2013) and more recently by other authors (Tomasova et al., 2019; Bouchalova and Bouchal, 2022; Ren et al., 2022; Hu et al., 2023); some are schematically resumed in Figure 2. The most straightforward approach used to investigate cell migration is possibly to plate cells on a culture dish compatible with live cell microscopy and to acquire images at fixed time intervals (Figure 2A). This method is effective in studying real-time cell movement and its changes, following both biological and chemical perturbations such as gene overexpression, silencing or drug treatments. It is relatively easy to set up by using state of the art microscopes and allows continuous observation of multiple fields of view in different samples. This setting is also extremely versatile and may be coupled with automatic liquid handlers, and, using automatic sample changers, image acquisition and analysis, can be significantly scaled up to include high throughput approaches (Patsch et al., 2016; Choi et al., 2021).

Time-lapse acquisition of cells randomly moving on a culture plate is a relatively simple approach, but may be easily coupled with other techniques, designed to systematically interfere with and specifically modify cell migration. Under agarose migration assays (Figure 2B), for example, consist of an agarose gel pad, polymerized on a glass, used to separate a cell population and a chemoattractant. A gradient is generated by diffusion of the chemoattractant into the gel so that cells, initially plated on a hole punched at the centre of the pad, will migrate under the gel towards the source of attractant placed at the gel periphery. The opposite geometry is also possible, with cells plated at the periphery of the gel pad, migrating towards the attractant placed at the centre. This assay provides a confined environment where cells can move adhesion dependently or independently and is particularly suited for studying migration of immune cells. It was first described in 1975 by Nelson et al. (Nelson et al., 1950) and has been used primarily to measure neutrophil, dendritic cells and monocytes chemotaxis, but also the chemotaxis of endothelial cells and *Dictyostelium discoideum* (Rupnick et al., 1988; Wessels et al., 1988; Stokes et al., 1990; Heit and Kubes, 2003; Song et al., 2006; Hadjout et al., 2007; Amselem et al., 2012; Schwarz and Sixt, 2016; Vargas et al., 2016).

Slightly more complex environments were obtained within migration chambers, where 3D scaffolds of collagen fibres were polymerized between two coverslips. In this type of setup, that partially recapitulates the dense microenvironment cells are experiencing *in vivo*, the ability of cells to contract their body as well as squeezing their nucleus, represents the limiting factor to advance within the matrix (Figure 2C) (Rommerswinkel et al., 2014).

Simple time-lapse microscopy has been largely employed also to study collective cell migration. It aims to investigate cell locomotion properties of the whole population taking in consideration also the impact of cell-cell interactions during motion. When cells reach high density on a suitable surface, streams of cells coherently moving in the same direction may appear in the monolayer (Figure 2D). In particular conditions, for example, when the small GTPase Rab5A is overexpressed in MCF-10A cells, the cluster of moving cells can reach the considerable length of 1 mm, roughly two orders of magnitude the size of a single cell (Malinverno et al., 2017). Similarly, in “wound healing” assays, a continuous monolayer may be used to study directional cell migration towards a cell free area produced by scratching the monolayer with a plastic tip or needle (Figure 2E) (Baumann, 2014; Grada et al., 2017). After the scratch, cells will polarise in the direction of the “wound” and, individually or collectively, migrate towards the now available empty area, eventually filling it up to restore the original monolayer. This assay is probably the simplest method able to mimic, *in vitro,* the repair process following a wound; it can be used to study the behaviour of a sheet of interconnected epithelial or endothelial cells, but also for populations of not connected cells such as fibroblast or other cell types. It has been widely used to study cell-cell and cell-ECM interactions during cell migration. Wound healing assays on culture plates have been used to demonstrate the role of cell adhesion molecules, Rho GTPases and mechanical forces in collective cell migration (Cai et al., 2014; Reffay et al., 2014; Das et al., 2015; Aoki et al., 2017). A scratch could be a rather traumatic event for the cells remaining on the newly formed border: the destroyed cells, in fact, release their intracellular content into the medium, possibly activating specific signalling pathways in the neighbouring cells. These side effects may be avoided, while standardising at the same time the manual scratch for different users, by means of alternative, less traumatic, methods to generate the cell-free area. A micro-stencil barrier placed within the culture plates, for example, prevents cell growth and, when removed, leaves a regular, well defined and width-controlled cell-free area, avoiding cell debris (Justus et al., 2014).

Another approach, to study directional cell migration, relies on devices such as the Zigmond chemotaxis chamber (Figure 2F), conceived to facilitate direct observation of single cell behaviour during chemotaxis. The observation area in the centre of the device connects two reservoirs which are filled with chemoattractant solutions at different concentrations. By diffusion of the chemoattractant a gradient is then formed in the observation area. Studies with this kind of assay allowed to distinguish chemokinesis, i.e., the general increase in cell speed, from cell directional bias, as early as 1991 (Zicha et al., 1991). Derivatives of the Zigmond chamber, as the Dunn and Insall chambers or the μ-Slide Chemotaxis device, improved control and longevity of the gradient up to 24 h or more. They are all based on direct visualisation of cells seeded in a low cross-section connector bridging two reservoirs of media at different chemoattractant concentration (Muinonen-Martin et al., 2010; Zengel et al., 2011; Malet-Engra et al., 2015). Malet-Engra and colleagues used the commercially available μ-Slide Chemotaxis to show how T lymphocyte directionally migrate as clusters displaying higher chemotactic sensitivity than as individual cells. The same device was later modified to enclose cells into a micro-fabricated arena; biased migration leads to the accumulation of cells on one side of the device, to evaluate the chemotactic effect without live cell imaging, considerably simplifying the experimental setting and substantially increasing experimental throughput. Tomasova and colleagues used the migration arena assay to study the chemoattractant activity of several growth factors on normal human epidermal keratinocytes (nHEK) (Sackmann et al., 2014; Tomasova et al., 2019). Another method to assess directional migration in response to a chemoattractant gradient is the Boyden chambers**/**transwell assay (Figure 2G). The assay relies on two chambers separated by a porous membrane through which a gradient is established; the top one is where cells are plated, while the bottom one is filled with attractant (or repellent) molecules such as chemokines, growth factors, lipids, nucleotides or other drugs. Cell motility is quantified by counting the number of cells passed through the membrane in a fixed amount of time. The assay duplicates as a sort of cell invasion assay, by adding extracellular matrix (ECM) materials on the membrane and seeding cells on top. This method measures both cell chemotaxis and capacity to pass through an extracellular matrix, a particularly relevant feature in the study of cancer metastasis formation or embryonic development (Castellone et al., 2011; Bouchalova and Bouchal, 2022). Moreover, by modulating the size of the membrane pores, the transwell assay allows to evaluate not only the ability of cells to follow a cue, but also their capacity to mechanically squeeze their body, and importantly their nucleus, the largest and stiffest organelle in the cell, through a narrow pore (Kalukula et al., 2022). These assays contributed to study the role of cell receptors and other modulators of cell migration such as small GTPases belonging to the Rho family (Castellone et al., 2011; Hall, 2012). “Transwell” chambers contributed to studying the role of cell plasticity, as well as the influence of extracellular matrix on cells migrating across a structured three dimensional medium (Cai et al., 2000; Pellet-Many et al., 2022).

Micropatterning of adhesive molecules on non-adhesive surfaces has been extensively applied in the study of cell polarity and cell migration (Figure 2H). By finely controlling adhesion area and then cell shape, this technique can partially recapitulate *in vitro* the complexity of the 3D cell micro-environment *in vivo*. Particularly, 1.5 μm in width lines, well mimic an oriented 3D fibrillar extracellular matrix (ECM) (Doyle et al., 2009; Théry, 2010). Similarly, wider lines, ranging between 5 and 20 μm in width, forcing cells in an highly polarised state, are able to recapitulate some features typical of 3D migration, such as Golgi and centrosome positioning, speed and directionality (Pouthas et al., 2008; Schuster et al., 2016). This experimental approach, by simplifying the problem of 2D cell trajectories analysis to 1D, allowed to unveil a common pattern over 50 different cell types, the correlation between cell speed and cell persistence, that is the ability of a cell to keep its direction (Maiuri et al., 2012). Indeed, these two parameters, in principle independent, are instead intrinsically correlated (Maiuri et al., 2015).

**FIGURE 3:**
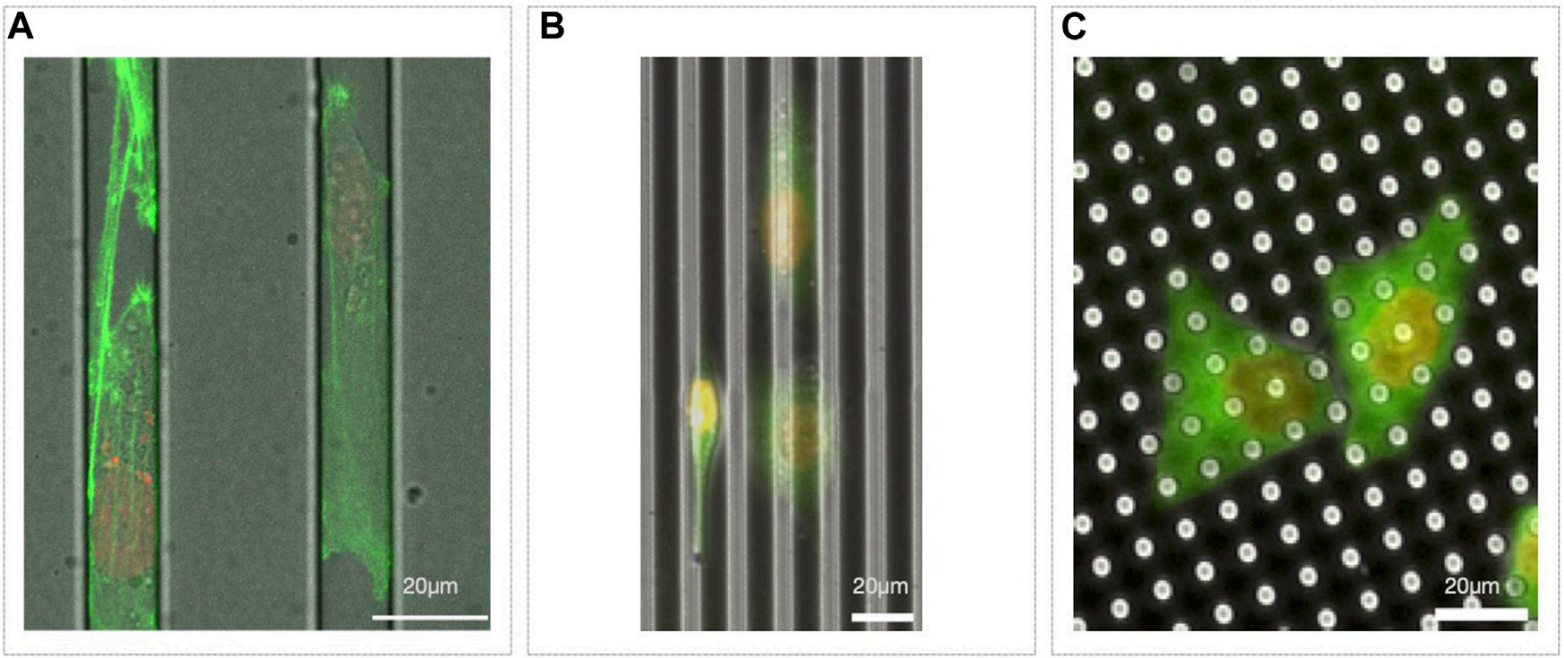
Microfabricated migration assays. **(A)** Microchannels with MDA-MB-231 Lifeact-GFP/H2B-RFP migrating inside; **(B)** 5 µm in deep groves with HeLa Tubulin-GFP/H2B-RFP on top, oriented according to the topography (some cells go at the bottom of the groves, see center right, while others stay on the top of it, see left); **(C)** pillars 7 µm high with HeLa Tubulin-GFP/H2B-RFP plated on top.

3D microfluidic channels could be considered a direct evolution of micropatterned lines of adhesive molecules (Figures 2I, Figure 3A). They have been employed in the study of migration of adherent and nonadherent cells, with and without a chemoattractant gradient or lateral constrictions (Li Jeon et al., 2002; Beta and Bodenschatz, 2011; Heuzé et al., 2011; Chabaud et al., 2015; Thiam et al., 2016). This kind of device, mimicking the complex environment migrating cells encounter moving in interstitial spaces, highlighted the complexity of this phenomenon. Indeed, observing cells moving in microchannels with tight constrictions, it has been elegantly shown how cells, moving in over-confined spaces, where they need to significantly squeeze their nucleus to go through, accidently induce nuclear envelope rupture and then consequent DNA damage (Denais et al., 2016; Raab et al., 2016).

To mimic *in vitro* the physical complexity of the *in vivo* micro-environment, further tuning of the cell substrates may be obtained by creating custom micro or nano-structured surfaces. Micro as well as nano-topography, indeed, strongly affects cell-substrate interaction, cell adhesion and then cell migration and, as example, cells tend to orient along micro or nano-groves (Figure 3B) (Refaaq et al., 2020). Similarly, cells also specifically react to surface roughness (Chighizola et al., 2022).

Microfabricated substrates can also be much more than just mere passive elements. Indeed, they can serve, as example, as specific force sensors. Cells plated on top of a forest of pillars of controlled stiffness would move deflecting the pillars proportionally to the force applied on it (Figure 3C). Since the deflection can be measured and the nominal stiffness of the material is known, it is then possible to estimate the local and total force a cell is exerting on the substrate while migrating (Schoen et al., 2010).

Although challenging for non-specialists, microfluidic-based migration assays are becoming more and more common, also thanks to different companies that developed specific tools to support cell biologists (Sackmann et al., 2014; Zhao et al., 2020; Milton et al., 2023; Shen et al., 2023; Yang et al., 2023).

Overall, bioengineering and material science allowed to open new perspectives in the study of cell migration and in understanding the underlying molecular mechanisms. Taking advantage of simplified systems, specifically designed to mimic *in vitro* some of the functional cues of the extracellular microenvironment, it is possible to quantitatively tune and then investigate the impact of many substrate properties, as: rheology, dimensionality, micro- and nano-topography or surface chemical functionalization. The simple modulation of the substrate mechanical properties, indeed, highlighted the crucial role of cellular tension on focal adhesions in cell spreading and migration. More generally, the temporal mechanical response of the extracellular environment, its rheological properties, together with cellular contractility, modulate the dynamic molecular stability of integrin clusters (Riveline et al., 2001; Geiger et al., 2009; Chaudhuri et al., 2020). This, then, altering the cell/substrate catch-bond process, clearly impacts on cell migration. Since integrin stability is mechano-mediated, and mesenchymal migration strongly relies on cell/substrate adhesion, a substrate with a stiffness gradient leads to biased migration. Interestingly, both possible cell behaviours have been observed. There are indeed cell types that preferentially move toward the stiffest substrate region, while others towards the softest. Generally, this process is called “durotaxis” (Barber-Pérez et al., 2020). Unfortunately, biomaterials are not ideal elastomers and their mechanical properties can not be recapitulated just by stiffness. Stress relaxation of soft viscoelastic substrate, indeed, has been proved to control a filopodia-mediated 2D migration mode possibly promoting cancer cell migration (Adebowale et al., 2021). Interestingly, the topography of the micro- or nano-structures on the surfaces, by physically shaping integrin clusters at cell-material interface, strongly affects cell migration by a process called “contact guidance” (Hsieh et al., 2018; Leclech and Villard, 2020; D’Urso and Kurniawan, 2020).

### Computational tools for evaluating motion features in experimental data

Experiments performed by acquiring images of moving cells over several hours and under different conditions, require further analysis to process primary data. Computational methods and tools are then essential to extract from cell migration experiments quantitative parameters describing cell motion behaviour such as speed, path linearity, average turning angle, time persistence or directional bias. They finally allow to describe motion of isolated single cells as well as cell monolayers or populations. Alongside the introduction of the various experimental setups, software packages and tools have been adapted to analyse different aspects of cell migration and quantify motion parameters. Additional packages have later been specifically developed to extract tracks from images and to analyse them.

Table 1 is a collection of tools developed to analyse monolayer dynamics, single cell migration or both. For each tool, the table reports basic information such as supported operating environments and availability (commercial packages and freely available ones were mentioned), the data types accepted as input and whether assisted cell tracking, evaluation of morphology and/or proliferation is provided. Regarding movement, the table includes tools supporting a broad range of movement analyses and modelling and that can present results using a variety of plots or other graphic representations (+++); also included are other tools providing a wide but less extensive range of features and analysis procedures (++), and some others that are specifically focused on the analysis of one or a few related migration properties (+). The table includes early packages, such as the recently discontinued Metamorph^1^, as well as applications, as CellProfiler and TrackMate, which underwent different revisions between their appearance and today, up to very recent options such as Migrate3D or LIM Tracker (Carpenter et al., 2006; Bray and Carpenter, 2015; Aragaki et al., 2022; Kinahan et al., 2023). Many are full applications, mostly locally running on commonly used desktop operating systems, but also remotely accessed through standard browsers or by other means; some tools work within specific environments such as ImageJ plugins, R^2^ or MATLAB^3^ packages (Downey et al., 2011; Meijering et al., 2012; Barry et al., 2015; Tinevez et al., 2017; Wortel et al., 2021; Aragaki et al., 2022; Ershov et al., 2022).

**TABLE 1:** List of free and commercial software packages evaluating cell movement.

Category	Tool	Year	Environment	Accepts as input/works on				Cell tracking	Evaluates			Availability
				Images	Coords	Cell layers	Single cells		Movement	Morphology	Proliferation	
General purpose image analysis package with support for movement analysis	Image-Pro	1991	Win	√		√	√	√	+			commercial
	MetaMorph	1993-23	Win	√			√	√	+++	√	√	commercial
	CellProfiler	2006-21	Win, Mac, Linux (Java)	√		√	√	√		√		free
	ImarisTrack	2009	Win, Mac	√	√		√	√	+	√	√	commercial
	Volocity Quantitation	2011	Win	√		√	√	√	+	√		commercial
	ilastik	2019	Win, Mac, Linux	√		√	√	√		√	√	free
Cell trackers with different amount of movement analysis	CellTrack	2008	Win, Mac, Linux	√			√	√	+	√		free
	TACTICS	2013	Win, Mac, Linux	√			√	√	+	√	√	free
	iTrack4U	2013	Win, Mac, Linux (Java)	√			√	√	++			free
	CellTracker	2016	Win, Mac, Linux	√			√	√	++			free
	CellMAPtracer	2021	Win, Mac, Linux	√			√	√	+		√	free
	TraCurate	2021	Win, Mac, Linux	√	√	√	√	√				free
Movement analysis with focus on cell paths	Cell-motility	2006	Win, Mac, Linux (Java)		√		√		++			free
	DIAS	2009	Mac	√			√	√	++	√		free
	MotoCell	2009	Web browser	√	√		√	√	+++		√	free
	Pathfinder	2013	Win (Java)	√		√	√	√	+++			free
	CellMissy	2013-17	Win, Mac, Linux		√	√	√		++			free
	celltrackR	2021	R		√		√		+++			free
	Migrate3D	2023	Win, Mac (Python)		√		√		++		√	free
Macro/plugin	TrackMate	2008-22	ImageJ	√			√	√	++	√	√	free
	MTrackJ	2012	ImageJ	√			√	√	++			free
	Chemotaxis & Migration	2019	ImageJ	√			√	√	++			free
	Adapt	2015	ImageJ	√		√	√	√	+	√		free
	LineageTracker	2011	ImageJ	√			√	√	+	√	√	free
	LIM Tracker	2022	ImageJ	√			√	√				free
	DiPer	2014	MS-Excel		√				++			free

Many of the described programs are essentially general-purpose image analysis packages (first group) which take a global approach, starting from raw data acquisition and including cell tracking and motion quantitation, although often to a limited extent. Tools included in this first group directly operate on the acquired images, to extract quantitative parameters related to cell position and status, like mitosis and division (MetaMorph^1^ and ImarisTrack^4^), or shape and morphology (Image-Pro^5^ and, more recently, ilastik) (Berg et al., 2019). Integrated image processing routines are typical of the programs in this group, as they can use segmentation procedures to separate stained cells or entire cell sheets from the background; these programs are effective when working on experimental assays oriented to characterise, by means of simple parameters, collective cell migration (see Figure 2 panels d and e) as well as movement of individual cells migrating on flat surfaces (Figure 2A,B,E,F), or also on micropatterned lines or 3D channels (h, i). The ability to study the migration of entire cell layers is particularly suited to wound healing assays (see Figure 2E), where migration behaviour is often quantified in terms of rate of edge advancement or of empty area filling. Many programs in this group are commercially available, with the notable exception of CellProfiler and ilastik, developed with an open source approach. The first, in addition to standard measurements (cell count, size, per-cell protein levels), allows more complex morphological evaluations (cell/organelle shape or subcellular patterns of DNA or protein staining), also in high throughput experiments. Taking advantage of an extensible architecture, CellProfiler uses plugin modules such as CellProfilerTracer to provide additional functionalities, such as support for visualisation and quality check of tracking data (Lamprecht et al., 2007; Bray and Carpenter, 2015). Within ilastik, several workflows are available, allowing image segmentation, object classification, counting and tracking; being able to work on both 2D images and 3D stacks, it can also be used in the analysis of experimental setups where cells are moving within a 3D matrix, such as within migration chambers depicted in panel c of Figure 2.

In experimental assays oriented to analyse single-cell movement on 2D surface as well as in 3D culture conditions, cell tracking is necessary to translate microscopy acquisitions into lists of coordinates describing the path followed by each cell. Manual identification of cell bodies, although often tedious and time-consuming, is still an effective and sometimes necessary approach, especially for phase contrast acquisitions and/or high-density cultures, where cells are not easily isolated from each other (Maška et al., 2023). To increase the dimension of experimental datasets, in more recent years a number of programs have appeared (second group in Table 1) which automate and optimise cell tracking using different types of images as input data. Most software tools provide automated cell tracking by detecting fluorescence-stained cell nuclei over a black background (for example, TACTICS, among others); some, like CellTracker and iTrack4u, can also identify unstained cells in images from bright-field or phase-contrast microscopy (Sacan et al., 2008; Wessels et al., 2009; Cordelières et al., 2013; Shimoni et al., 2013; Piccinini et al., 2016; Ghannoum et al., 2021). Within this group, all tools are equipped with procedures useful to address basic movement analysis issues such as visualization of cell trajectories, quantitation of speed and direction of movement, area coverage, cell deformation. Still in the second group, TraCurate focuses on connecting cell detection/segmentation with automated tracking, manual correction and finishing and can also import tracking data produced by a variety of other established tracking software. Although not including analysis options, it allows a large number of analyses by linking raw motion data with movement analysis tools provided by the R environment (Wagner et al., 2021).

Programs in the third block in Table 1 are specifically focused on motion investigations and include tools able to provide very in-depth analyses. Some programs, such as CellMotility and CellMissy, completely skip the tracking procedure and only work on previously collected cell trajectories. The first is noteworthy for the broad support for the analysis of single cell motion in two dimensions, in experimental setups such as those depicted in panels a, b, e, f of Figure 2, but also in 2h and 2i, where cells are forced to move along a straight trajectory. It is an open-source application, able to compute many motion parameters such as trajectory MSDs, persistence time and cell motion speed; in addition it includes movement model testing (Martens et al., 2006). CellMissy, initially designed to analyse wound healing assays (panel e of Figure 2), later added a database to support comparative analysis of multiple experiments. It supports multi-parametric (speed, directionality), multiscale (step, trajectory), and quality-controlled analyses and allows fast comparison across different tested conditions, also providing data visualisation and assisted data filtering (Masuzzo et al., 2013; Masuzzo et al., 2017)*.* Dynamic Image Analysis System (DIAS) extracts quantitative data about instantaneous velocity, direction of travel, direction change and chemotactic index (Wessels et al., 2009). Migrate3D was recently proposed as a tool to evaluate a large number of parameters starting from datasets produced by many different image processing tools also with the help of Principal Component Analysis (PCA) (Kinahan et al., 2023). This group also contains some movement analysers able to address complex experimental questions by providing a wide range of analyses and parameters. Among them, MotoCell which, unlike others, is a web application, was designed to study many aspects of 2D individual cell motility, including dynamic image analyses and visualization, cell tracking and statistical analysis of cell behaviour. It also supports the evaluation of descriptive parameters, calculated for the whole population as well as for each individual cell (Cantarella et al., 2009). Pathfinder investigates both individual and collective cell migration: starting from fluorescence microscopy data, it computes migration parameters, such as instantaneous and time-averaged migration speed and direction, as well as direction change frequency. Additionally, it characterises collective cell migration by quantifying, for each cell, number of neighboring cells and average standard deviation of their migration angles (Chapnick et al., 2013). Although not an application, CellTrackR, a package available within the R environment, contains an extensive set of tools for both 2D and 3D cell trajectories analysis. It was designed to calculate several motion parameters and provides, in addition to data management and quality control, methods to extract and visualise cell migration features and specific algorithms for trajectory clustering. Motion of immune cells migrating under specific experimental conditions such as under agarose or migration chambers (Figures 2B,C) can be easily explored with this tool (Wortel et al., 2021).

In addition to the previously described tools, a number of plugins designed to work within ImageJ/Fiji^6^ add cell tracking or other movement-related features to the environment and may be used as steps within customized workflows (Gorelik and Gautreau, 2014). LIM Tracker, possibly the simplest in this group, allows to effectively track cell bodies producing paths that may be analysed with other tools. TrackMate (Tinevez et al., 2017) offers automated and semi-automated tracking algorithms for fluorescence microscopy images, together with additional visualization and analysis tools; in its latest release (2022) (Ershov et al., 2022), it can even interface with external tools, such as ilastik (Berg et al., 2019) and cellpose (Stringer et al., 2021), to add advanced segmentation procedures. Adapt was designed to study membrane morphodynamics by automated detection of protrusions and filopodia and provides quantitative data on cell morphology, membrane dynamics, changes in protrusion size and/or fluorescence staining at the cell periphery (Barry et al., 2015).

A number of open source algorithms and procedures not yet mentioned, although not included in the table, also deserve a note as they originated from the collaboration of researchers operating in different and independent fields and resulted in very promising tools, versatile and adaptable to different needs. In the field of image processing, Scikit-image is a Python tool which provides collections of algorithms such as skimage, able to perform complex morphological analyses or volumetric measures on multi-dimensional images (Walt et al., 2014). Napari^7^ comes from an interesting experience of a completely open group of contributors interacting with the project and is an interactive viewer for multi-dimensional images, with annotation, processing and tracking tools. Deep learning approaches of course contributed to increase the calculation power needed to manage and analyse complex image collections and translate their contents in numerical features. As an example, cellpose successfully uses deep learning to segment images and can be used with datasets of cells imaged with different microscopy and labelling techniques (Stringer et al., 2021).

### Main numerical parameters used to describe migration features

The results of time-lapse acquisitions and cell tracking procedures may be analysed to quantitatively estimate collective and/or single cell migration parameters. Collective analysis of cell migration includes the evaluation of the degree of plate surface coverage, expressed as percent confluence, or progression of the cell front, as in wound healing assays. To evaluate single cell migration, cell positions are tracked and analysed to calculate parameters that describe cell displacement, whose length can be used to calculate mean speed, path length, i.e., total distance travelled by a given cell, net displacement, i.e., the distance between start and end point of a path, linearity, defined as the ratio between path length and net displacement.

Most of the software packages described in Table 1 analyse movement features by evaluating speed, motion persistence and directionality, even though often they refer to them using different denominations and definitions. Persistence, represented as a tendency to maintain, at each time step, the previous direction, has been variably referred to as persistence, linearity or sometimes also directionality, and differently measured in time units, i.e., how long the current direction influences the movement in subsequent time periods, or in terms of ratio between net displacement and length of the followed path. What is called persistence within AveMap (Deforet et al., 2012) and iTrack4U (Cordelières et al., 2013) is named linearity in MotoCell (Cantarella et al., 2009) and end-point directionality ratio in CellMissy single-cell module (Masuzzo et al., 2017). Pathfinder (Chapnick et al., 2013) describes persistence in terms of the absolute angle of deflection, while in Cell_motility (Martens et al., 2006) persistence is expressed in time units and calculated, as proposed by Alt et al., in 1988 (Othmer et al., 1988), using the model initially described by Fürth et al., in 1920.

Overall, the analysis of multidimensional datasets produced by innovative techniques generates data which, although certainly contribute to the field by providing immediate information, still contain a hidden level of knowledge, more difficult to access, that could be unlocked by integrating and mining primary data. The availability of all these data and analysis tools creates new challenging opportunities but poses some problems; one of them is certainly the shared definition of analysis parameters as well as the standardisation of experimental protocols and data formats. Some attempts in this direction are underway; a standard format for cell migration tracking files, for example, has been provided by Biotracks^8^, which also provides a set of converters able to translate files from other popular tracking software packages to the biotracks format. Another interesting initiative is the Cell Migration Standardisation Organisation (CMSO), started in 2015 with the mission to develop shared standards in the field of cell migration, and still active in supporting the cell migration research community by providing a long-term open data sharing environment (Gonzalez-Beltran et al., 2020). Created within the scope of CMSO, MIACME^9^ (Minimum Information About Cell Migration Experiments) is a document, a sort of guideline, that lists essential elements for the minimal description of cell migration experiments. In the currently available release (v 3.0), the document provides three main sections to describe experimental setup data, imaging setup, and raw, as well as processed, images dataset obtained.

## Conclusion

Locomotion is an ancestral and essential property of cells, from unicellular to multicellular organisms. In live organisms cells migrate in different situations, such as during tissue morphogenesis, immune response, wound repair or cancer spreading.

Essentially cell migration may be described as a physical process where reciprocal feedback connect mechanical properties of cells and substrate, chemical composition of solid and liquid micro-environment components, and signalling networks driven by intracellular molecular changes. It is a plastic and dynamic process where cell migration features rapidly change as a function of different external stimuli. To unveil its fundamental mechanisms, the process need to be drastically simplified, to decipher it by separately studying single components of this intricate phenomenon. In this work we review many established classical experimental methods used to investigate *in vitro* cell migration on 1, two and three dimensions, and provide a compilation of “state of the art” computational tools used to quantitatively interpret experimental results. Most methods rely on observing cells while moving on the surface of untreated or modified culture dish, and/or while moving through chemical gradients. These methods allow to finely evaluate the ability of individual cells, or cell monolayers, to sense and react to environmental changes. The introduction of micro-fabricated devices highlighted the determinant role of mechanical constraints as well as physical properties of cell substrates, such as stiffness and viscoelasticity, or surface nano-features, on cell locomotion. Direct tracing of cells moving within live organisms or in tissues is desirable although quite challenging due to limited transparency of samples. Nevertheless, live imaging has been achieved recording fluorescent cells movements in the fin of live medaka fishes (Maiuri et al., 2015), or also imaging the development of entire model organisms, such as chicken or *drosophila* embryos (Dai and Montell, 2016; Yoshihi et al., 2022). Some recent approaches allow to infer cell migration by extra-cellular matrix or basal membrane tracks deposited by migrating cells (Chen et al., 2017).

Images of cells moving *in vitro*, but also ex or *in vivo*, represent primary data waiting to be translated into numerical parameters to obtain a quantitative description of motion behavior. Computational methods and tools play an essential role in this phase to extract tracks from images and to analyse them. The reviewed tools and procedures were developed and/or adapted to extract tracks from images and analyse them to quantify motion parameters. Mathematical conceptualization significantly helped to interpret cell migration data and the correct definition and quantification of parameters describing cell locomotion properties allowed the design of models able to accurately describe cell locomotion behavior (Toscano et al., 2022). We envisage the development of elaborate *in silico* systems that, by integrating the vast knowledge of molecular pathways together with cell mechanical properties, will generate predictive models of cell movement. This approach will certainly help to produce a more comprehensive picture of cell locomotion.

## Footnotes

1 D.S. Galileo and V.P. Patel, “FOCUS: Tracking Migrating Cells with Metamorph^®^ Software.”

2 R: The R Project for Statistical Computing; https://www.r-project.org

3 MATLAB: “MATLAB”; https://www.mathworks.com/products/matlab.html

4 Imaris for Tracking; Oxford instruments https://imaris.oxinst.com/products/imaris-for-tracking

5 Image-Pro | Advanced Image Analysis’ 2023; https://mediacy.com/image-pro

6 ImageJ Wiki https://imagej.github.io

7 Biotracks: https://pypi.org/project/biotracks/

8 MIACME:http://cmso.science/MIACME/v0.3/#abstract

9 Napari: https://napari.org/stable/
